# How paediatricians investigate early developmental impairment in the UK: a qualitative descriptive study

**DOI:** 10.1186/s12887-022-03233-1

**Published:** 2022-05-16

**Authors:** Mark Atherton, Anthony R. Hart

**Affiliations:** Department of Paediatric Neurology, Sheffield Children’s NHS Foundation Trust, Ryegate Children’s Centre, Tapton Crescent Road, Sheffield, S10 5DD UK

**Keywords:** Child Development, Developmental Disabilities, Etiology, Diagnosis Differential, Investigative Techniques

## Abstract

**Background:**

Early developmental impairment (EDI) is common and has many aetiologies and, therefore, potential investigations. There are several published guidelines recommending aetiological investigations, and paediatricians’ views of them varies. Little is known on the thought processes underlying clinical decisions in investigating EDI. This study aimed to describe the thought processes affecting clinical decisions on the investigation of EDI within a nationalised health care system.

**Methods:**

A qualitative descriptive study using semi-structured qualitative interviews performed in person or via video link with paediatricians who see children with EDI in England. As part of the interview, a case study of a fictional disease, Cavorite deficiency, modelled on biotinidase deficiency, was given to participants with the cost of testing, incidence and likelihood it would respond to treatment. This allowed exploration of cost without encumbrance from predisposing views and training on the condition. Thematic analysis was performed by iterative approach. Where participants stated they wanted to redirect money from investigations to treatment, were that even possible, we asked which services they would like to be better funded in their area.

**Results:**

Interviews were conducted with 14 consultant paediatricians: 9 Community / Neurodisability, 2 General paediatricians, and 3 Paediatric Neurologists. Two themes were identified: the value of an aetiological diagnosis to families and managing risk and probability when investigating EDI. The latter contained 4 subthemes: ‘circumspection’ involved blanket investigations chosen irrespective of phenotype and high regard for guidelines; ‘accepting appropriate risk’ involved participants choosing investigations based on clinical phenotype, recognising some aetiologies would be missed; consultants found they ‘transitioned between practices’ during their career; and ‘improved practice’ was thought possible with better evidence on how to stratify investigations based on phenotype. Services that were most frequently reported to need additional funding were therapy services, early community developmental services, management of behaviour, sleep and mental health, and educational support.

**Conclusions:**

There are many factors that influence paediatricians’ choice of aetiological investigation in EDI, but clinical factors are the most important. Paediatricians want better evidence to allow them to select the right investigations for each child without a significant risk of missing an important diagnosis.

**Supplementary Information:**

The online version contains supplementary material available at 10.1186/s12887-022-03233-1.

## Introduction

Early developmental impairment (EDI) is a group of conditions manifesting when a child’s developmental abilities lie two or more standard deviations below the population mean in at least two developmental domains [1, 2]. EDI affects 2–3% of young children [1, 3]. The Office of National Statistics mid-year population statistics for 2019 estimate there are 3,857,263 children under 4 years of age in the UK; [4] therefore EDI will affect around 77,000–116,000 children and between 20,000–25,000 will present each year. EDI can be divided into two broad groups: isolated EDI (EDI-) is where there are no other clinical features in the phenotype other than the developmental issues, whilst EDI + is where there are additional features present, such as macrocephaly, microcephaly, dysmorphia, organomegaly, abnormalities of movement or muscle tone, eye abnormalities, seizures, consanguinity, or a relevant family history [5]. Paediatricians are more likely to investigate EDI + than EDI- [6]. There is variation in paediatricians’ views on the usefulness of specific investigations and whether or not children are investigated appropriately [6]. A number of guidelines exist for investigating EDI, but they are rarely evidence based, are often based on consensus, and the recommended investigations vary significantly [1, 3, 7–21]. In 2017, we reviewed the frequency of diagnostic investigations for EDI in our centre and recommended rationalisation of investigations, with the consideration for cost-savings one potential benefit of this approach [5, 22]. These recommendations were controversial, with other groups arguing in favour of additional testing [23].

Whilst there is large variation in what investigations paediatricians request in children with EDI, there is no published evidence on how paediatricians choose aetiological investigations for EDI. Understanding why paediatricians choose the tests they do would identify internal and external driving factors, and could aid professional groups in reaching consensus about how to investigate EDI, particularly in the age in which greater genetic testing is being introduced. For example, it is tempting to assume that genetic testing through exome or genome testing will become the only first line test in EDI, but similar hopes about microarray have not come to fruition, [5] and understanding motivations for testing are important if investigations are to be rationalised in the future.

This study took place in the UK and the context of the health service is important. The UK has a nationalised health service, in which clinicians have freedom to choose investigations and treatments according to clinical need, other than for highly expensive and specialised conditions, without having to consider costs to the hospital or patients / family or involve insurance companies. On occasions, there may be pressures from hospital managers or the government to reduce costs and improve efficiency, but the degree to which clinicians feel this pressure will vary, and the ultimate decision on what a patient needs remains with the clinician. For children with EDI, referrals may be made to several specialists: general paediatricians, community paediatricians, paediatricians with expertise in neurodisability, or paediatric neurologists. Although a proportion of paediatric neurologists only see children with EDI via referral from other paediatricians following initial assessment and investigation, other neurologists take referrals direct from primary care. The preliminary training for all UK paediatricians is identical, with at least 5 years of general paediatric training, followed by additional training in sub-specialisms or general paediatrics to reach consultant level. Therefore, the initial training and experience of investigating EDI is the same across sub-specialisms, and there is likely to be significant concordance in views.

This qualitative descriptive study aimed to describe the thought processes and factors that affect UK paediatrician’s decisions on investigation choice in children with EDI, including the cost of testing.

## Methods

### Participant identification

Our inclusion criteria were consultant paediatricians who routinely assess and investigate children with EDI in England. We included general, community, neurodisability, and neurology paediatricians to gain the fullest range of views on clinical practice. Although this would allow us to collect a range of opinions, the harmonised basic training and culture within the health care system means we expected a high degree of homogeneity in views. We had no limit to the maximum number of paediatricians we would recruit and were mindful of guidelines on thematic saturation.[24, 25] We initially aimed to recruit 12–15 participants, but found we reached data saturation at 11 participants. We recruited another 3 to ensure no further codes arose and to ensure a balance of expertise across participants.

We identified a list of potential participants by sending an email to paediatricians in UK community paediatric units inviting them to participate, and we asked them to forward it to other paediatricians involved in the assessment of children with EDI. We also advertised the study in the weekly newsletter of the British Paediatric Neurology Association (BPNA), whose membership includes paediatric neurologists, neurodisability specialists and allied specialties. We approached paediatricians who were published in this field, including those known to disagree with our previous work and suggestions, to capture the full spectrum of views. We also used a snowball technique, i.e. by asking participants to identify additional potential participants. From this convenience sample, we used purposeful sampling. We started by recruiting participants who were published in the field and had contrasting views to our own. Following this, we recruited equal proportions of community, general, neurodisability paediatricians and paediatric neurologists, men and women, a range of duration of consultant experience, and from a wide geographical area of England. No more than 2 participants were recruited from any single city.

### Topic guide / questions

A topic guide was written based on the findings of our previous work, [5, 6, 22] with an aim to discover how participants choose when to investigate children with EDI, how they choose which investigations to choose, their views on specific investigations, and to explore how much costs affected their choice of investigation. The topic guide (supplementary material, available online) commenced with open questions on their job, factors in the history and examination that lead to investigation, their attitude to departmental guidelines, followed by questions on specific tests. We followed these questions by presenting them with the cost of each test at our hospital, and exploring how this information changed their views, if at all. Finally, we wanted to assess how much the cost of a test affected decision making unencumbered by the participants’ experiences of the usefulness of specific tests in their clinical practice or training. To do this, we gave participants a case study of a fictitious condition called Cavorite deficiency, named after a mineral found in an HG Wells novel. Cavorite deficiency was modelled on biotinidase deficiency, which some clinicians state can present with EDI-, [14] and included the same incidence figures [26] and costs [22]. The final question presented the likely cost across the UK to diagnose one participant with Cavorite deficiency and whether they thought this was good value for money. If not, we asked how they would prefer to spend this funding in their clinical practice by listing all services they wish they had more access to. We counted the frequency each service was mentioned across all participants. The topic guide was trialled in 3 volunteers and revised to remove duplicated questions and clarify ambiguity.

### Interview methodology

A single interviewer performed the semi-structured interviews (MA). The interviewer was a male senior paediatric neurology trainee near consultant level studying for an MSc in Child Health. The number of consultant and trainees in paediatric neurology are small in the UK, so the interviewer may have known or worked with the interviewee previously. The interviewer had previously published in the area of EDI, and may have had preconceived views that led to bias. We reduced this risk by ensuring the topic guide included open questions that were not leading, and via training on how to conduct interviews. The interviews were arranged at a time and location of the participants’ choice and were performed 1:1 where possible. When the COVID-19 pandemic began, interviews were performed virtually via video link. Written informed consent was obtained from all participants.

### Data analysis

The interviews were recorded digitally, transcribed verbatim, anonymised, and checked for accuracy. Thematic analysis was performed as per Braun and Clarke (2006) [27]. This included familiarisation of data, initial coding using an inductive approach by two researchers (MA and ARH), review of initial codes, agreement on a coding structure for the whole dataset, and identification of a thematic structure to determine main and subthemes. Themes were developed using an iterative process to capture all range of views. NVivo for Mac version 12 (QSR International PTY Ltd, 2018) was used for analysis. We provide illustrative quotations for evidence of our results, and triangulated our findings with previously published data in this field, as presented in the discussion section. Ethical approval was obtained from the University of Sheffield (Reference Number 029999).

## Results

Fourteen health care professionals were interviewed (Table 1). Two interviews were terminated early, one because of a meeting and another because of technical difficulties with the video link. Both were completed within 7 days. The duration of interviews ranged from 48 to 97 min (median 76 min).

**Table 1:** Characteristics of participants

Participation	Location	Speciality	Time since completion of specialist training
1, Female	1, Yorkshire and Humber	Community / Neurodisability Paediatrics	> 20 years
2, Male	1, Yorkshire and Humber	Community / Neurodisability Paediatrics	> 20 years
3, Female	2, South East England	Community / Neurodisability Paediatrics	10–20 years
4, Female	3,Yorkshire and Humber	Community / Neurodisability Paediatrics	< 5 years
5, Male	2, South East England	Paediatric Neurology	< 5 years
6, Female	4, Yorkshire and Humber	Community / Neurodisability Paediatrics	< 5 years
7, Female	5. Midlands	General Paediatrics	5–10 years
8, Male	5. Midlands	General Paediatrics, Neurology / Neurodisability	10–20 years
9, Female	6. East of England	Paediatric Neurology	< 5 years
10, Female	3, Yorkshire and Humber	Community / Neurodisability Paediatrics	> 20 years
11, Female	7, North England	Community / Neurodisability Paediatrics	> 20 years
12, Male	8, North England	Community / Neurodisability Paediatrics	> 20 years
13, Male	9, North England	Paediatric Neurology	10–20 years
14, Female	10. Yorkshire and Humber	Community / Neurodisability Paediatrics	5–10 years

**Fig. 1:**
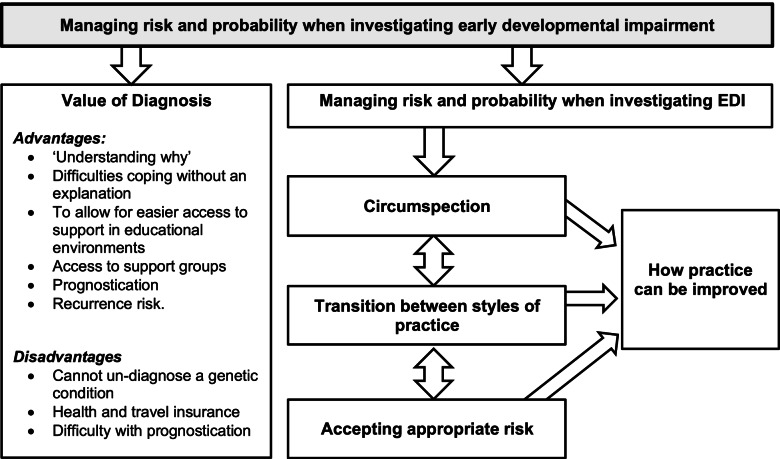
Summary of results of thematic analysis from qualitative interview study

Two major themes emerged during analysis (Fig. 1):

“The value of an aetiological diagnosis’”

“Managing risk and probability when investigating EDI”, from which the following subthemes were found:

- *“Circumspection”*
- *“Accepting appropriate risk”*
- *“Transition between styles of practice”*
- *“How practice could be improved”*

**The value of an aetiological diagnosis**—illustrative quotations are shown in Table 2.

**Table 2:** Illustrative quotations for Theme 1 – The Value of an aetiological diagnosis

Subject	Quotation	Participant
Why an aetiological diagnosis is important in EDI	*“I think sometimes families have real difficulties in not having an answer as to why their child has those difficulties”*	6
	*“I think it’s fair for the families if they want to know and need to know and, in most cases in my experience, they do—the why, the how, the what, and the what next…Also some of the time it allows you to remove the burden of guilt upon a family and allow them to, in some cases, they might begin to grieve, and support them through that process in a more supportive way.”*	8
	*“There may be the opportunity to, um, provide, share with the family expectations of the pattern that we might see evolving over time, allow them to come to terms with the diagnosis, receive relevant advice or relevant support, perhaps input from um whatever clinician specific associations and support groups, and to be able to explain it better to the other family members and friends.”*	8
	*“I think understanding the nature of the beast is a massively important thing for parents. To think about the future, to think about how to prepare for the future, and if we could get as much information that can help them do that, then all the better.”*	7
	*“[Families] want to know the reason why their child has got their difficulties, particularly when they’re arranging school as well, because they will ask them what their difficulties are and sometimes schools don’t always recognise early developmental delay as being an actual diagnosis, and they really don’t understand, well, why they have this, when actually a lot of the time we just don’t know.”*	6
	*“I mean some of these conditions are horrendous neuro-metabolic conditions and you would want to give the family opportunity to think about whether or not they want to have further children or do antenatal testing.”*	13
Disadvantage of aetiological diagnosis	*“…You can’t de-diagnose a genetic diagnosis and, erm, I'm very aware that in some parts of the world if you do have a genetic diagnosis, you don’t get health insurance.”*	1
	*[relating to a diagnosis giving prognostic information] “I think for some of those families it can be really hard as well because they are expecting something to happen and, if it doesn’t happen in the timescales, then they can find that really distressing as well.”*	6

Consultants recognised the value to parents of an aetiological diagnosis for EDI, including: ‘understanding why’; the difficulties coping without an explanation; to allow for easier access to support in educational environments where the diagnosis of EDI was not accepted; access to support groups for specific conditions; prognostication; and recurrence risk. One participant highlighted a number of disadvantages to an aetiological diagnosis, particularly for ethnic minorities, including the inability to “take back” a genetic diagnosis, the negative effect on health and travel insurance, and how accurate prognostication could not be guaranteed because of phenotypical variation of genetic conditions.

**Managing risk and probability when investigating EDI**—illustrative quotations are shown in Table 3.

**Table 3:** Illustrative quotations for Theme 2—Managing risk and probability when investigating EDI

Subject	Quotation	Participant
**Subtheme 1: Circumspection**		
Performing a battery of tests	*“We have a set form on ICE [investigation requesting software] for all developmental delay, so we can tick all of those… it almost kind of feels as if there is a button that you just press and you don’t have to remember which investigations to do. So, I think, perhaps in a way, we have almost got lazy in that we just do that rather than thinking clinically.”*	6
Guidelines provide safety and standardised practice	*“I think some of my colleagues do like to have a guideline that they will just follow because that makes them safe.”*	3
	*“I guess the reason why I'm a guideline person is, erm, if somebody wanted to question my practice of why I'm doing it I just, it’s a comfort blanket to say, ‘Yep, I'm always following those guidelines. I wasn’t practising as a maverick!’”*	1
	*“I think the, err, advantage of having a national or international guidance is that, err… if you’ve followed it, err, you have, err, greater, you know, medicolegal immunity, if you like, to being challenged. Whereas if you have deviated, then you are at risk.”*	2
Justifying practice by reducing unpleasant procedures	*“It’s better for children to have [investigations] all in one go….if the child needs numerous needles, it’s not a particularly nice experience.”*	6
**Subtheme 2: An acceptable level of risk**		
An acceptable degree of risk	*I remember as a trainee being told years ago that you should always feel slightly anxious that you might have missed something because, if you don’t, then you are over-investigating.”*	3
	*“We have to acknowledge that, um, we won’t always be right, and to have the humility to accept that we will sometimes be wrong, within the caveat of the fact that there are other support measures around to guide the practice.”*	8
Over-investigation as a problem	*“I think at the moment we perhaps over investigate in terms of the numbers. I think it is okay to investigate children but I think we probably need to be a bit smarter.”*	6
The difficulties presented by guidelines	*“I think guidelines give people um the ability to cop out of the situation and they need to think.”*	5
	*“I think guidelines are really important, but they are more useful to less specialist people um and to trainees. Now that might sound a bit patronising, and I don’t mean it like that, but as you are moving around specialities in paediatrics you don’t know everything about everything.”*	10
The need to over-ride a guideline	*“So, whether a test is within a guideline I suppose would have some sway on individuals and would have some sway on me, provided I’ve got the flexibility to um over-ride that if I feel that, actually, at that point in time, either it was not warranted or there was something about the child that would make me, umm, want to deviate from it.”*	8
The importance of clinical phenotype	*“My approach tends to be that I would, err, come at it from, erm, from what I like to think of as their biopsychosocial risk factors or I do a risk stratification in my head.…I would go through the history in great detail and the examination in great detail, and within the history and the clinical examination, I would be looking for specific risk factors which are pointing me towards any particular areas of difficulty.”*	2
	*“I think there is no point being a doctor, you might as well have a robot, if you’re going to do everything straight away. It’s about clinical acumen, isn’t it?”*	7
	*“There’s a paper that a biochemist from X did … and if you looked at that, you’d go bananas because there’s so many metabolic conditions and I’ve got to pick up everything?! I don’t think so! And that’s why it’s important that the clinical examination is so, so very important, and taking the history, and clinical skills are important. You can’t just do a tick box exercise. No!”*	12
The stepwise approach to investigation	*“I would think about have we looked for an underlying cause to a reasonable extent. So that would be the first level of investigation. If that hasn’t yielded a cause, a second level of investigation. After a second level of investigation that I consider to be thorough, then I would usually stop investigating. I may refer the child to a geneticist, if I think that is relevant.”*	9
	*“I still feel more comfortable having covered the basics knowing liver, kidneys and thyroid are basically working, the body is working okay, before I start exhibiting any medicines of any kind.”*	11
Exceptions to the stepwise approach	*“… particularly the children with ASD, a blood test is quite, can be quite abusive.”*	10
	*“The only time I would think of doing more tests… say, if a child were to have MRI brain under general anaesthetic, I would think carefully if the child needed a lumbar puncture for CSF neurotransmitters and, of course, then I would want to do them both together at the same time. But, no, otherwise it’s step-wise approach.”*	14
Invasiveness and inconvenience of investigations to families	*“I think we do need to think about the invasiveness of a test. I think that you know they’re not nice procedures in children and it is very quick for us, because we don’t have to do it, we don’t see how distressed the children get, we just write on a form and send them off to somewhere else to have it done.”*	4
	*“Because our children are seen in community clinics they need to go to blood tests…for blood tests, they have to go to the hospital. Erm, so it is a trip for the parents and, you know, paying for parking, and all the rest of it.”*	3
Watch if initial investigations are negative	*“One of the other tests that we’ve not talked about that I think is under rated is time, and that is re-visiting after an interval to see what’s changed and not, you know, not forgetting you can do that. Because, sometimes children grow into a condition and it is more obvious later on.”*	11
Parental influence	*“I would take into consideration where parents and the family are. And there are some families for whom the anxiety, err, doesn’t let them function and it affects their mental health, err, everything else. And in those situations, sometimes, following a discussion, I will go for a particular investigation which they are completely over-focussed on.”*	12
Inconvenience to clinicians of false positive or insufficient samples	*“The more investigations you do, the more you pick up false positives, the more you stress everyone out, the more blood you end up needing to take, the more, you know ermm—and it’s costly. And actually, you know, if we send everything on everybody we end up making a lot of work for us and hassle to the families.”*	3
Over-investigation	*“That comes again to the over investigating, you could do harm to a child by doing that.”*	13
Missed diagnoses	*“I think people tend to go with what they have been taught and I think if either they’ve seen something that’s been missed, or they’ve been trained by somebody who has seen something that has been missed, I think that tends to raise people’s level of concern and make them more likely to do more tests.“*	4
Paediatric Neurologists	*“Paediatric neurology sees one end of the spectrum. You know, you don’t have clinics full of children who have nothing other than developmental delay. You only see the children with lots of other exciting stuff going on, who were much more likely to have a diagnosis that you can make.”*	3
	*“I wonder if that’s partly because people have… parents have a certain expectation, the referrer has a certain expectation, it’s very difficult for a neurologist to see patient um and not add something to the investigations that have already been done, because you sort of feel that’s what’s been expected of you when the person has made the referral.”*	9
The influence of cost of investigations on the health service	*“I think it’s really low yield and I just, I just, I just think we’re a national health service and we need to think about costs, and I know a lot of people don’t want to think it’s appropriate for doctors to even be involved in that at all, but I don’t think that… that’s life. We do have to think about costs. There are services we cannot get, such as there is no community services at all available for our ASD kids or, you know, and maybe if we didn’t do so many first line really expensive things that are really unlikely to yield results, then we would have more money for other things.”*	7
Participants don’t know the cost of investigations	*“The cost of the test, err, we don’t have a recent idea of what the cost of the tests are.”*	12
Cost is less important if the condition is treatable unless it’s very rare	*“As a consultant, umm, I’ve sort of reflected upon, more the treatable aspects that I can do in neurology and I try not to miss those things and because, as I said before the… if I can…, that one child that I picked up, you know, his life changed, but that also saved the NHS somewhere between £5 m—£40 m. So, I would happily do, for the rest of my career, just for very long chain fatty acids, acylcarnitine and biotinidase, without worry that I am going to bring the NHS to its knees.”*	5
	*“If it was treatable to the point of retrieving somebody’s cognitive function fully, you know, these things would be in my mind. If there is a possibility of doing a fantastic treatment, even if it was a very rare condition, I would be prepared to spend a lot more.”*	9
Newborn screening	*“If it’s a treatable condition, there would need to be a discussion about whether it should be included in the newborn screening programme.”*	11
Clinicians cannot make these choices on a population level about cost	*“For me, they are the kind of decisions that NICE [National Institute of Clinical Excellence] get paid all the money to make.… I don’t think those kind of decisions are mine to make. I don’t think that’s for an individual clinician.”*	4
Redirecting money from investigations to other services	*“I mean there’s so much more that we could spend that on if we weren’t wasting it on investigations that weren’t necessary and weren’t telling us the right things! Then that could go into more physio, more speech and language, more interventions for the children that have got developmental delay…”*	4
	*“We may never see this money would we really? (Laughs). I think that’s what it comes to, because you would not see this £6 m. It won’t be in your budget. It won’t be in a place where you can use to develop your services.”*	12
**Transition between styles of practice**		
Relying more on phenotype with clinical experience	*“I have to admit that I’m changing in my approach to neurological presentations in general and probably in developmental delay as well, and that err in the past, when I was a young paediatric neurologist, I was probably quite aggressive and, you know, you do lots of tests. And now I am a bit more thinking, ‘um do I have to do that straight away or do I wait and see?’ and actually there’s so much to gain from monitoring things over time… I’m probably sometimes a little bit more hesitant to do invasive tests.”*	13
	*“As I’ve gone along my journey of many years in the health service I think I’ve cut down the number of tests that I did. You know, historically, I might have done more metabolic type tests on everybody, but actually the yield was low and I learned that from auditing my practice and realising that actually I’m not picking up much here, there’s a better way to do it, and try and keep up with the literature.”*	11
**How practice could be improved**		
Evidence to produce a national guideline that stratifies investigations according to phenotype	*“I know that’s what the Sheffield paper said, like, ‘Well, what’s the point because you pay so much money and you don’t get feedback?’, but actually reading the paper the children weren’t investigated consistently with that, with a battery. So, you couldn’t actually answer that question for the very rare conditions. So, if we had a regional or a national cohort where we did a certain test for a child with isolated developmental delay, we could finally say, ‘you know, actually we found nobody doing biotinidase, so let’s stop doing it’ or actually you might find we had two patients and we should really be doing it… or maybe we could say, ‘Well, actually we’ve got two patients but they were both of Asian origin, so, in Caucasians we really can forget about doing this test, but in other ethnicities we should’. And I think, because we don’t have that population approach, we haven’t been able to get the answer. So, if we had an agreement about a set of tests we do, and do that for a period of time, we might get some of that back and that would be just so amazing.”*	13
Collaboration with others	*“I’m very lucky because I do a joint clinic with my neurology colleagues and I’ve done it for all the time I’ve been working here and we are really fortunate because we learn from each other’s experience.”*	12
	*“I do a monthly clinic with my colleagues in the regional clinical genetics service. It’s something, you know, we talk about things in every clinic and … I learn from his experience and the regional experience, and I’m always open to changing my practice because what I want to be is efficient and effective, but not be over-using investigations that are going to cause worry to families and that are going to be costly to undertake.”*	11

This theme, exploring the factors influencing choice of aetiological investigations, contained a spectrum of attitudes, at the end of which were two distinct approaches: ‘circumspection’ and ‘accepting appropriate risk’. Most participants were *“in both camps”*, and had changed the degree to which they ascribed to each attitude as they gained clinical experience, which formed our third subtheme, *“Transition between styles of practice”*.

*“Circumspection”* is the practice of avoiding taking risks and being cautious in one’s behaviour. In the context of this study, this related to requesting a large number of tests to ensure no aetiology was missed. Participants described both their own and other clinicians’ practice in which a large number of investigations were requested without reference to clinical features or the likelihood they would be diagnostic. This was driven by computerised investigation processes in some cases, where a pre-determined set of investigations were ordered. In this subtheme, guidelines and standardisation of practice between colleagues and centres were highly valued, and provided protection from both medico-legal claims and criticism if a diagnosis was missed.

*“Accepting appropriate risk”* In this subtheme, participants acknowledged they “won’t always be right” and an aetiological diagnosis may be missed. The key was to ensure this risk was acceptably small. Over-investigation was viewed negatively and guidelines were considered to be too simplistic to cover the full complexity of EDI, did not place enough emphasis on clinical phenotype or the prevalence of conditions in local populations, and led to under or over-investigation. If participants ascribing to this subtheme did use guidelines, they reserved the right to over-rule them by adding or subtracting tests they thought clinically appropriate.

Clinical phenotyping based on history and examination was highly valued. Participants preferred to adopt a stepwise approach to investigation, performing the tests most likely to reveal an aetiology first, possibly alongside a “general health screen”, then moving to second or third line testing where initial tests were non-diagnostic. There were two exceptions: children with an autistic spectrum disorder, where venesection would be distressing or impossible, and where a child required an MRI under general anaesthetic. The invasiveness of the test and the inconvenience and cost to the family of attending hospital for the investigations were considered important. Where initial investigations were non-diagnostic, clinical ‘watching and waiting’ helped to identify new ‘clues’ as the child grew older and enable access to new investigations when they became available. Parents influenced the choice of investigations if there was anxiety about specific conditions or investigations that affected their mental health.

False positive results or insufficient samples leading to repeated tests were an inconvenience that created additional, unnecessary work, and increased the child and families’ inconvenience. A low yield for investigations was considered “over-investigation”. Previously missed cases, either by the participant or someone that trained them, led to a focus on that particular investigation in the future.

The speciality of the consultant played a role: community and neurodisability paediatricians thought neurologists ordered more tests, which paediatric neurologists accepted. Neurologists reported a strong focus on the diagnosis of rare conditions and were more likely to request metabolic investigations, but noted they saw more complex children and felt there was an expectation from the families and referrers to perform some additional investigations to justify the referral. Phenotyping remained important to neurologists.

There was a complex relationship with the cost of investigations. It was suggested phenotyping led to rationalisation of investigations, and was justified as ‘cost efficient”. However, only one participant reported pressure from managers to reduce expenditure, participants did not know the cost of aetiological investigations, and all reported they would order a highly expensive test if they had a strong suspicion of the condition. The degree a condition was treatable was important, but was balanced against the incidence of the condition, rather than cost. For example, most participants would not order serum Cavorite if the incidence of the condition was extremely low, even if the cost was small. Neurologists were more likely to request Cavorite, but two changed their view when the cost was over £1000 a test, and suggested it should be on newborn screening instead. They also noted the health economic savings of improving a small number of children’s outcome may be greater than the cost of testing a large number of children. Although all participants agreed cost was important, they frequently deferred decisions on a population level to national governmental bodies. Participants agreed in principle that it would be good to redirect money saved on unnecessary investigations to fund improved access to therapeutic interventions they needed in their unit (Table 4), but noted this was unrealistic within current health service structures.

**Table 4:** Other services for which participants would like to see more provision

Other services participants want greater access and funding directed towards
*Therapy services and other allied health care services*
• Speech and language therapy (7 participants) • Physiotherapy (5 participants) • Occupational therapy (5 participants) • Wheelchair services (1 participant) • Neuropsychology (1 participant) • Dietician (1 participant)
*Community early intervention services*
• Portage / parenting support to promote child development in early years (6 participants) • Health visitors (2 participants)
*Mental health / behaviour support*
• Behavioural management services (5 participants) • Sleep services with expertise in children with abnormal development (5 participants) • Support for children and families with an autistic spectrum disorder (4 participants) • Child and Adolescent Mental Health services / Child and Adolescent Psychiatrists (3 participants) • Learning disability support / nurses (3 participants)
*Educational services*
• Educational support services (2 participants)
*Other areas of clinical practice*
• Ability to perform assessment with all professionals together as a multidisciplinary team (3 participants) • EEG / neurophysiology (1 participant) • Respite care (1 participant)

*“Transition between styles of practice”* – this subtheme described how practice changes with greater experience and seniority. Participants reported they had changed their style of practice as they gained experience, moving from circumspection to accepting appropriate risk. Important factors driving this behaviour change were experience of a high number of false positive or ambiguous results, following audit or reflection of their experience of non-diagnostic results, and following training and new evidence on specific investigations and conditions.

*How practice could be improved –* this subtheme explored how paediatricians thought either their own or their colleagues’ practices could be improved or standardised, including how they managed their anxiety and received reassurance that their practice was appropriate and no diagnosis had been missed. Participants valued team meetings to peer review cases, and greater access to advice and joint clinics with other specialities, such as neurologists, metabolic teams, and geneticists. Further research was recommended to provide evidence on which investigations were useful according to phenotype, which would allow for a nationally agreed, evidence-based guideline on the stratification of investigations.

## Discussion

Paediatricians recognise the value of an aetiological diagnosis in EDI for families, and cited many of the same reasons that parents do: validation, information, procuring services or therapies in the education setting, early intervention, support, need to know, and prenatal diagnosis [28]. Many published guidelines recommend clinical phenotyping to choose which investigations are first and second line [1, 3, 5, 7–21]. Previous qualitative data collected from 107 UK clinicians and published in 2017, showed 61% had departmental guidelines for the investigation of EDI and 62% of responders thought children with EDI + were investigated the right amount, compared to 38% children with EDI- [6]. Amongst our participants, 79% had departmental guidelines, and 50% thought guidelines investigated children the right amount, suggesting our data is valid and reflects the range of views on EDI.

We have previously suggested that reducing the burden of unnecessary tests could produce cost savings to the health service, and found the yield of metabolic investigations was low, particularly in EDI- [22]. This agrees with other groups’ work [1, 5, 14, 29–31]. However, other publications extol the importance of diagnosing metabolic disorders, [20, 21, 23, 32–34] particularly where they are treatable. Such divergence in views was also seen amongst our participants. Community / neurodisability and general paediatricians found the yield of metabolic investigations was low and recommendations for large numbers of routine metabolic tests were described as “bananas”! These interviewees reported problems with routine screening of metabolic conditions, including the large volumes of blood needed and the inconvenience to children and families of repeat testing when samples were insufficient, falsely positive, or of uncertain significance. Over time, many of our consultants dropped routine metabolic investigations because the yield was so low. Cost efficiency was highlighted in our previous work as a potential advantage of reducing investigations requested in EDI, [5, 22] and interviewees themselves cited this as one reason for reducing the number of metabolic tests they ordered. However, it was hard to establish cost as a behavioural modifier from our data, despite what was said: participants did not know the cost of investigations, rarely faced cost pressures from managers as a negative reinforcement, and did not experience a positive reinforcement of having saved money redirected to interventional services. There was also a suspicion money would redirected to other acute hospital services instead, perpetuating what one participant called the “Cinderella status” of community paediatrics. What is more likely is that consultants change their behaviour because of either the positive punishment factor of the additional work created by spurious results, or negative reinforcement from never finding a positive result.

In comparison, neurologists and more inexperienced paediatricians were more likely to investigate rare treatable metabolic conditions because they were treatable, but acknowledged many of these would be better investigated on newborn screening to allow for early diagnosis and treatment before developmental impairment occurred. Despite this divergence in views, all participants retained a belief that phenotyping was essential to aid with the choice of appropriate investigations. The differences between interviewees holding these two views related only to the degree of risk for missing a treatable condition considered acceptable. With phenotyping seen as important, guidelines were noted to be unable to cover every clinical situation, but were useful in making trainees and inexperienced consultants feel safe, particularly around rare conditions.

The criticism we faced when recommending reducing amounts of investigations in EDI guidelines [23] was valid in many ways, including the observation that not every child in our cohort received the same investigations. In reality, we think the actual difference in opinions is not so large: our data suggested little evidence for a large battery of tests in EDI-, which was where rationalisation of investigations was recommended, but we encouraged further testing in EDI + based on phenotype [5]. Given only 20% of our cohort had EDI-, this was the minority of children. Therefore, any disagreement primarily relates to how children are stratified based on clinical assessment and the acceptable level of risk for missing diagnoses. There is no good evidence to help with such stratification, and our participants all wanted a high-quality, evidence-based guideline according to clinical phenotype. Participant 13 suggested a large cohort study in which children with EDI were rigorously phenotyped and offered the same investigations, allowing evidence to be collected on which investigations are useful in different phenotypes. Such studies are both difficult to design and expensive, and we have been unsuccessful on a number of occasions to obtain funding for similar research projects.

In the near future, the availability of exome studies may also be a driver to change practice. A recent meta-analysis showed 31% of children with EDI- and 53% with EDI + had genetic abnormalities on exome studies, [35] so suggestions that exome studies become the only first line investigation may arise, with additional investigations reserved to determine the biochemical significance of variants of uncertain significance or for acquired, non-genetic causes. However, a number of observations suggest this may not be how paediatricians will practice in reality. Firstly, microarray was supposed to be introduced in our unit as the only first line investigation, with subsequent tests performed in children with negative results. Instead, microarray became yet another investigation in a long list of investigations. This is the recommendation in published guidelines, [3, 19–21] suggesting our local experience is not unique. Secondly, our data suggests interviewees may perform additional tests, either because they are performing venesection to obtain DNA and wish to reduce trauma to the child and inconvenience to the family, or because they wish to arrange a general health screen for other modifiable health factors. Therefore, we should not assume that, even if this is how geneticists think exome studies should be used, paediatricians will behave in this way.

The strength of our data includes collecting the views of all types of consultants who see children with EDI, the open nature of our questions, and discussing the cost of investigating rare diseases by using a fictional condition, which removed preconceived ideas about specific diseases. The use of qualitative interviews allowed much richer exploration of this issue than we had managed previously via qualitative questionnaires [6]. There are also limitations to our data. As with any qualitative data, the findings may not be generalisable, but we purposefully chose clinicians from different specialities and geographical regions to obtain a full range of views. Our data is also in concordance with our previous qualitative data from a national survey [6]. We reached data saturation so our sample size was appropriate. The COVID-19 pandemic meant interviews had to be performed virtually midway through recruitment, and we do not know the impact of this on our data. The interviewer was junior to the participants interviewed, and this disparity may have played a role, alongside any previous working relationship he had with interviewees. He may also have preconceived opinions prior to the study, but we mitigated these factors in study design and training. Finally, the attitudes of clinicians in a nationalised health care system are likely to be different to other health care systems, so this data may not be applicable outside the UK.

In conclusion, paediatricians acknowledge the importance for families of finding an aetiology for EDI, and this is not restricted to treatable conditions: the “need to know why” is recognised as important. When choosing investigations, paediatricians fall into two broad groups: the first is where a large battery of tests is applied to all children investigated with no consideration of clinical phenotyping or likely differential diagnosis. Standardisation of practice and guidelines are highly valued. These paediatricians tend to be the least experienced and eschew risk taking. The second are those whose choice of investigations are based on the clinical phenotype and an acceptable level of risk is acknowledged. These paediatricians tend to be more senior and have transitioned from a low risk, standardised approach towards accepting some risk as they have gained clinical experience. The cost of tests played only a small part in the decision-making process, mainly because any savings in expenditure were unlikely to be reinvested in other services, such as therapy or psychology, so there was little motivation to change behaviour. Decisions on cost tended to be deferred to national or governmental bodies.

What this study adds

- More inexperienced paediatricians have greater likelihood to request investigations without phenotyping to avoid missing treatable diagnoses
- Experienced paediatricians typically tolerate an acceptable risk of missing diagnoses
- Consultants transition from cautiousness to accepting risk during their career
- Factors influencing transition include non-diagnostic tests, false positives, invasiveness, and treatability
- Evidence is required to stratify choice of investigation based on phenotype

## Supplementary Information

**Additional file 1.**

## Data Availability

The data from the qualitative interviews are not publicly available to maintain confidentiality of centres and individuals, as per ethical approval. However, all reasonable requests for information will be provided on request to the corresponding author.
