# Supplementary material for: How paediatricians investigate early developmental impairment in the UK: a qualitative descriptive study
Source: BMC Pediatr. 2022 May 16;22:285. doi: 10.1186/s12887-022-03233-1 (PMC9109194; doi:10.1186/s12887-022-03233-1)
Supplement: Supplementary file 1 — Additional file 1. [file 12887_2022_3233_MOESM1_ESM.pdf]

What influences clinicians' choice of investigation in EDI?
Interview Schedule

Things to do before the start of the interview:

- Introduce yourself and explain you are a paediatric registrar studying for a Masters Degree in Child Health. You are learning more about how doctors investigate EDI (also called global developmental delay)
- Ensure you are somewhere quiet with minimal distractions
- Check the interviewee is happy to continue with the project
- Ensure they know you will record them on the dictaphones
- Ask them to sign and initial (NOT X) the boxes on the consent form
- Start both Dictaphones

General opening question

Can you tell me more about your job and what situations you might see a child with EDI?

Current practice

How do you investigate children with EDI?

- When do you choose to investigate a child with EDI?
- Do you routinely investigate all children you see with EDI? If no, ask – what are the things that make you to choose to investigate a child with EDI?
- What things in the history or examination influence which tests you choose?
- Does your department have a guideline? If yes, what do you think of it? What makes you deviate from your guideline? Do guidelines encourage people to investigate the right amount?
- How much does missing a diagnosis affect your choice of investigations?
- Is it better to do lots of tests in one go to be kind to the child or use a stepwise approach, with the most common or important causes first and other tests later if the initial tests are negative?

I would like to ask you about specific tests or groups of tests now -

- What are your views on performing a "metabolic screen" in all children with EDI?
- What are your views on performing a lead level in all children with EDI?
    - Have you seen lead toxicity causing EDI?
- What are your views on biotinidase levels in children with EDI?
    - Have you seen biotinidase deficiency causing EDI? What were the features?
- Which children do you do MRI in? How useful is it in your experience?

How much do the costs of tests affect your decision?

- One of the most commonly used guidelines, the MacDonald guidelines (see sheet), can cost around £400 per child. What are your views on this cost?
- It is estimated there are around 23,000 children each year presenting with EDI. If we investigated all of them using first line tests in the MacDonald guidelines, it could cost the UK £9.2million. How does that affect your views on costs?
- If we did MRI under sedation or GA in all children in the UK with EDI, then the cost to the NHS is between £25 to 30 million. How does that affect your view on costs?

What influences clinicians' choice of investigation in EDI? Interview Schedule

- Can you think of any health services that children with EDI would benefit from which are not currently available? [examples - sleep services, psychology, nurse specialists]
- If we could divert money from doing investigations towards these services, would that change which investigations you do?

We wanted to talk a little bit more about when a test is "too expensive"

Please read this clinical vignette. Your local guideline recommends a list of tests, including one called Cavorite. Cavorite deficiency is a treatable cause of EDI.

- Should we test for Cavorite deficiency?
- Cavorite testing costs £50 per child. What do you think about the cost?
- How would your views change if the cost of the test was £200?
- How would your views change if the cost of the test was £2000?
- How does the fact it is treatable affect your decision?

The incidence of Cavorite deficiency is estimated to be 1 in 130,000.

If we had 23,000 children per year with EDI, that would mean we diagnose one child with EDI every five years. It would cost £6.5million to diagnose one child.

- Is that worth the money? Why do you say that?
  - If no: shouldn't we investigate for the condition because it is potentially treatable?
  - If yes: some people report that there other services which are badly funded and could really help lots of children with EDI, like sleep services or psychology. If we can't fund both, which is better – to diagnose and treat one child with Cavorite deficiency them, or miss the rare diagnosis and help lots of other children?

- Please see this table. How much is too much (i.e. where is the limit) to diagnose one child presenting with EDI with Cavorite deficiency?

Closing questions

- What would help the uncertainty about the best way to investigate EDI?
- Is there something else that you think we should have discussed about investigating EDI but haven't?

All questions should be about the interviewees experience and their stories.

- Tell me about…
- What is your experience of?
- I'm interested in….
- Can you explain more about…

What influences clinicians' choice of investigation in EDI?
Interview Schedule

McDonald Guidelines for investigating EDI

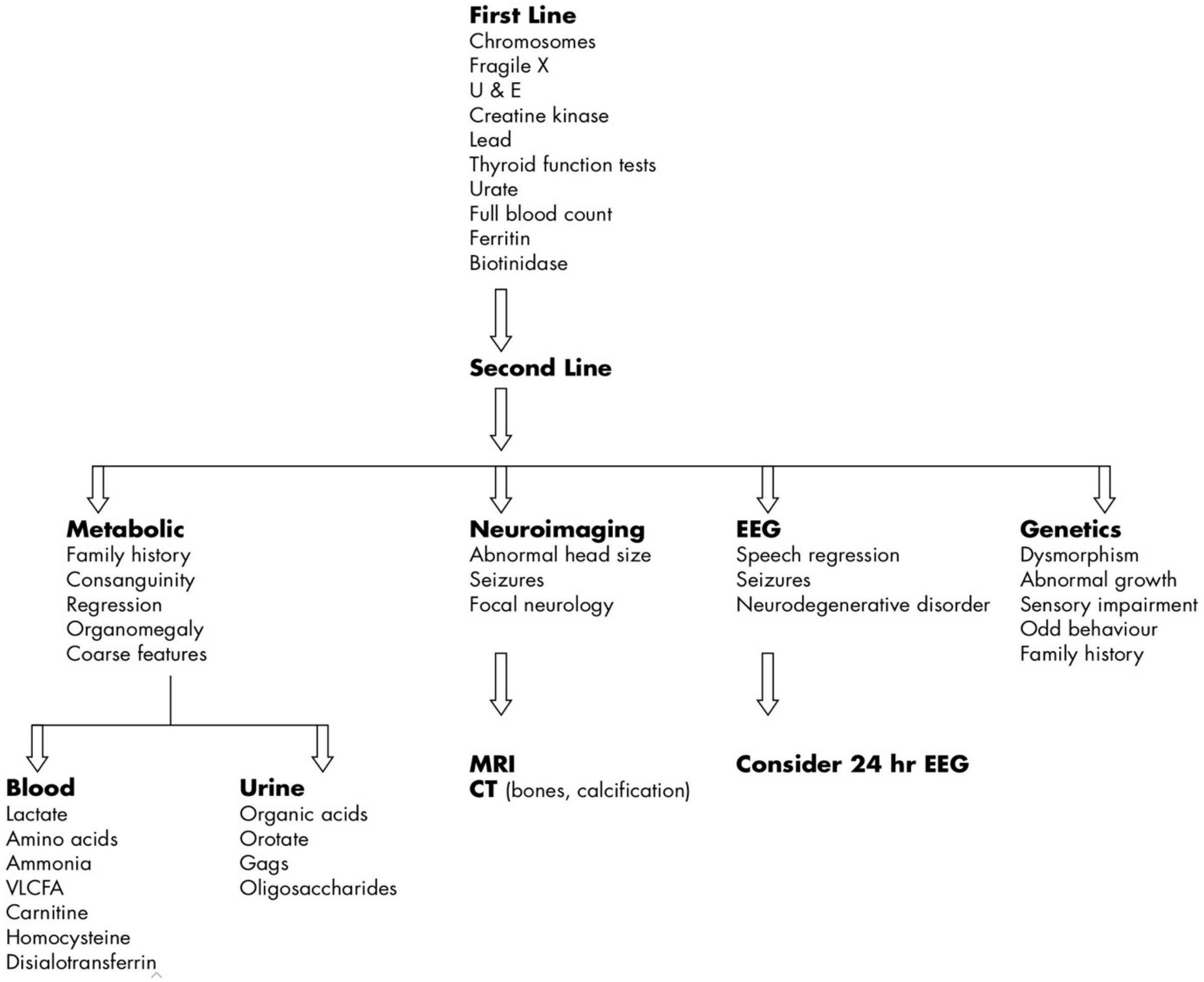

What influences clinicians' choice of investigation in EDI?
Interview Schedule

Case study

You see a 3 year old boy whose mother is worried about his development. When you see him, you think his development is around the 15 months level.

On examination, he has a head circumference that is large, around the 91st centile, and height and weight around the 25th. There are no dysmorphic features, and otherwise he looks well.

There are no other relevant features on examination.

Your local guideline suggests the following first line tests (other tests such as metabolic tests are second line):

- *CGH Microarray*
- *Full blood count*
- *Ferritin*
- *Renal Function*
- *Liver function*
- *CK*
- *Thyroid function test*
- *Plasma Cavorite*

Plasma Cavorite is a (fictitious) test for Cavorite deficiency, which causes Early Developmental Impairment.

If you diagnose Cavorite Deficiency, you can provide treatment and improve the child's outcome, although he / she may not return entirely to the normal range.

What influences clinicians' choice of investigation in EDI?
Interview Schedule

How much is acceptable amount of money to spend to diagnose one child with Cavorite Deficiency?

None – it's so rare or expensive we should not test for it
£1000
£5000
£10,000
£50,000
£100,000
£250,000
£500,000
£750,000
£1,000,000
£2,500,000
£5,000,000
£7,000,000
Just spend whatever it costs because it's treatable
It's just impossible to answer this question
